# Practical Departments

**Published:** 1905-11-18

**Authors:** 


### PRACTICAL DEPARTMENTS.

### MESSRS. JAMES HOWORTH AND COMPANY'S ARCHIMEDEAN SCREW VENTILATOR.

This is an apparatus, made by Messrs. James Howorth and Co., of Farnworth, near Bolton, Lancashire, which, it is claimed, will perform the same work, by the action of any wind that may be blowing, that is performed by a fan or a heating apparatus in producing an exhaust current in any room to which the ventilator is attached. It is also claimed that the apparatus will cure smoky chimneys. Over 250,000 Archimedean screw ventilators are stated to have been made at Messrs. Howorth's works. The apparatus consists of a pipe connected to the chimney, or upper end of the exhaust duct, or to any opening in the upper part of the room to be ventilated, that is arranged to carry off the vitiated air in the room, above the tube being a guard outlet for the air or smoke, the guard being of stout bars; above the outlet is a hood formed of vanes, a fan in fact, suspended on a vertical spindle, supported from the pipe itself by side brackets within an ornamental mounting above, The spindle which carries the upper fan, or vanes, also carries a lower fan fixed below the air outlet, formed on the lines of an Archimedean screw. The upper and lower fans revolve together whenever any wind is present, but it is claimed that the arrangement of the vanes of the upper fan and of the Archimedean screw are such that the net result is a pumping or exhausting action, similar to that of a suction fan, and also that no down draught is possible. The whole apparatus is constructed of galvanised sheet steel, except the hood, which is made from three-cross tin, and the spindles which are made from the best bar steel, the whole being balanced on knife edges, so as to revolve with very little pressure of wind, and constructed, the makers state, to stand wear for a good many years. They claim that the exhausting effect is due to the exact angles of the Archimedean screw being adhered to.

